# Reevaluating Authorship in the Age of AI: The Case Against ChatGPT as a Scientific Co-Author

**DOI:** 10.34172/aim.34214

**Published:** 2025-07-01

**Authors:** Masoud Keikha

**Affiliations:** ^1^Tropical and Communicable Diseases Research Center, Iranshahr University of Medical Sciences, Iranshahr, Iran; ^2^Department of Medical Microbiology, School of Medicine, Iranshahr University of Medical, Sciences, Iranshahr, Iran

## Dear Editor,

 The advent of advanced language models such as ChatGPT has sparked significant debate regarding their role in scientific research and authorship.^1^ We concur with the perspective that while artificial intelligence (AI) holds utility in scientific workflows, its limitations demand careful ethical scrutiny. In this correspondence, we aim to elucidate the fundamental challenges and ethical considerations associated with attributing scientific authorship to AI.

## Authorship Criteria and AI Limitations

 The International Committee of Medical Journal Editors (ICMJE) outlines four essential criteria for authorship: substantial contributions to the conception or design of the work; drafting or revising it critically; final approval of the version to be published; and agreement to be accountable for all aspects of the work.^2^ ChatGPT, as an AI system, lacks cognitive intentionality, legal accountability, and the ability to engage in ethical reasoning. It cannot provide critical analysis, endorse intellectual content, or assume responsibility for published material. Assigning authorship to such a tool undermines these established norms and erodes the accountability framework that underpins scientific trust.

 Recent evaluations underscore AI’s inadequacy in generating verifiable, domain-specific scientific content. Gao et al demonstrated that AI-generated abstracts may appear superficially plausible yet often contain conceptual inaccuracies and lack of analytical depth.^3^ Similarly, van Dis et al showed that ChatGPT is prone to generating fabricated references and unverifiable claims, highlighting serious concerns about information integrity.^4^ These observations collectively reinforce the conclusion that while AI may support human authors, it cannot replace them in fulfilling the authorship criteria.

## Ethical Implications and the Risk of Misconduct

 The inclusion of AI as a named co-author raises profound ethical and epistemological concerns. Central among these is the inability of AI to assume moral or legal responsibility for the accuracy and originality of published content. This raises the specter of untraceable accountability and undermines transparency in the research process.

 Moreover, there is increasing awareness of AI’s potential to unintentionally reproduce biased, plagiarized, or ethically problematic content. A recent study revealed that ChatGPT-generated text can closely mimic published academic material, potentially breaching norms around originality and copyright.^5^ The covert use of AI tools without proper disclosure further compounds this risk and may constitute a form of academic misconduct, particularly when misrepresented as the intellectual product of human authors.

## Editorial and Policy Responses from Leading Medical Journals

 In response to these challenges, several high-ranking international medical journals have issued explicit policies delineating the appropriate use of AI tools in manuscript preparation. These policies collectively affirm that AI does not meet authorship standards and must be used with transparency and caution.

 The Lancet has emphasized that “AI tools do not meet the requirements for authorship and cannot be credited as authors.” It further mandates that any use of such tools must be disclosed, and ultimate responsibility must reside with human authors.^6^ Similarly, the BMJ has asserted that “AI tools such as ChatGPT cannot be listed as authors,” reiterating that responsibility for accuracy, interpretation, and originality rests solely with human contributors.^7^

 Nature, in a 2023 editorial, firmly stated that large language models cannot be credited as authors and that any use of such tools must be reported transparently in the methods or acknowledgments section.^8^ These rules are consistent across all Nature Portfolio journals. In parallel, *JAMA* introduced policies requiring disclosure of AI use while explicitly excluding AI from authorship, encouraging clarity on how these tools contributed to the manuscript.^9^ Science has adopted a similar stance, forbidding the use of AI-generated content without explicit approval and full disclosure, citing risks to originality and scientific integrity.^10^

 This alignment across prestigious journals highlights a shared understanding: while AI can augment human productivity, it cannot satisfy the intellectual, ethical, or legal prerequisites of authorship. These editorials reinforce the distinction between tool and thinker, underscoring that scientific authorship must remain a human responsibility.

## Recommendations for the Scientific Community

 To safeguard scientific rigor and maintain ethical standards in the age of AI, we propose the following:

1. Institutionalized Policies on AI Use: Academic journals and research institutions must adopt and enforce comprehensive guidelines delineating acceptable AI usage. These policies should prohibit authorship attribution to AI and define the scope of permissible AI assistance.
2. Mandatory Disclosure of AI Involvement: Authors should be required to disclose the use of AI tools, including their specific functions (e.g. language editing, grammar correction), to ensure transparency and allow editorial evaluation of potential influence.
3. Education and Oversight Mechanisms: The research community should be educated on the strengths and limitations of AI tools. Oversight structures should be established to detect and deter improper or misleading uses of AI in manuscript development.

While AI systems like ChatGPT can enhance productivity and linguistic clarity, they remain tools devoid of intellectual autonomy, ethical awareness, and legal accountability. Recognizing their utility must not lead to conflating assistance with authorship. To preserve the credibility and reliability of scientific literature, it is essential to reassert human responsibility as the cornerstone of authorship.
